# Dupilumab and the potential risk of eosinophilic pneumonia: case report, literature review, and FAERS database analysis

**DOI:** 10.3389/fimmu.2023.1277734

**Published:** 2024-01-08

**Authors:** Xiyuan Zhou, Ge Yang, Xuemei Zeng, Lan Wang, Jing Xiang, Jinyu Zhao, Xuejun Chen, Lixia Zhang

**Affiliations:** ^1^ Institute of Dermatology and Venereology, Sichuan Provincial People’s Hospital, University of Electronic Science and Technology, Chengdu, China; ^2^ Respiratory Department, Chengfei Hospital, Chengdu, China; ^3^ Operation and Management Department, Sichuan Provincial People’s Hospital, University of Electronic Science and Technology, Chengdu, China; ^4^ Outpaitent Department, Sichuan Provincial People’s Hospital, University of Electronic Science and Technology, Chengdu, China; ^5^ Education and Training Department, Sichuan Provincial People’s Hospital, University of Electronic Science and Technology, Chengdu, China

**Keywords:** dupilumab, eosinophilic pneumonia, risk analysis, case report, FAERS database

## Abstract

Eosinophilic pneumonia (EP) is a rare but noteworthy adverse effect linked to dupilumab, an interleukin-4 (IL-4) and IL-13 inhibitor used in the managing atopic diseases. The underlying mechanisms, potential predisposing factors, clinical characteristics, and optimal management strategies for dupilumab-induced EP remain unclear. We report a 71-year-old patient who developed acute EP after the first 600-mg dose of dupilumab. Eosinophils (EOSs) were also transiently increased (up to 1,600 cells/μl). After the acute EP was effectively treated with glucocorticoids, dupilumab treatment was continued. Rash, itching, and immunoglobulin E levels continued to decrease in the patient, and no further pulmonary adverse events occurred. We combined this case with a literature review of nine articles and analyzed data from 93 cases reported in the FDA Adverse Event Reporting System (FAERS) database of patients developing EP after dupilumab use. Our findings imply that dupilumab may induce EP, particularly in individuals over 45 years old, those with a history of respiratory diseases, and those who have previously used inhaled or systemic steroids. Vigilance is required, especially when there is a persistent elevation in peripheral blood EOSs during treatment. Although steroid treatment can effectively manage EP, more data are needed to determine the safety of resuming dupilumab treatment after controlling pneumonia.

## 1 Introduction

Dupilumab, a monoclonal antibody that targets the interleukin-4 (IL-4) receptor alpha subunit (IL-4Rα), has emerged as a novel therapeutic agent for moderate to severe atopic dermatitis (AD). This innovative drug effectively inhibits the signaling pathways of both IL-4 and IL-13, which are key cytokines involved in AD pathogenesis (1). Dupilumab administration has been associated with significant clinical improvement in patients with AD, negating the need for routine laboratory monitoring (2–4). Its safety profile is also noteworthy, with minor adverse events (AEs) such as conjunctivitis, upper respiratory tract infections, and injection site reactions being commonly reported (5).

However, we report an unusual case of acute eosinophilic pneumonia (EP) in a 71-year-old patient with AD, which developed 6 days after dupilumab initiation. Upon extensive literature review, we found 12 additional cases of EP following dupilumab use for various conditions including asthma, rhinitis, AD, and pemphigus. EP, a rare disease characterized by cough, dyspnea, chest pain, and fever, can be induced by secondary causes like drugs and vaccines (6–8). Severe cases can lead to serious hypoxemia and pose a significant life threat (9). Although our case is not the first instance of EP induced by dupilumab, it adds to the growing body of literature suggesting a potential link between dupilumab and eosinophilic lung diseases. Given its rarity and potential severity, it is crucial to revisit these cases to better understand the relationship between dupilumab and EP.

Alongside this case study, we have also examined the FAERS database for further reports of EP following dupilumab use. Our aim is to identify patient groups that may require more vigilant monitoring during dupilumab therapy and explore potential mechanisms underlying these AEs. Through this effort, we hope to enhance the application of dupilumab, balancing its therapeutic benefits against potential risks.

## 2 Case presentation

**Figure 1:**
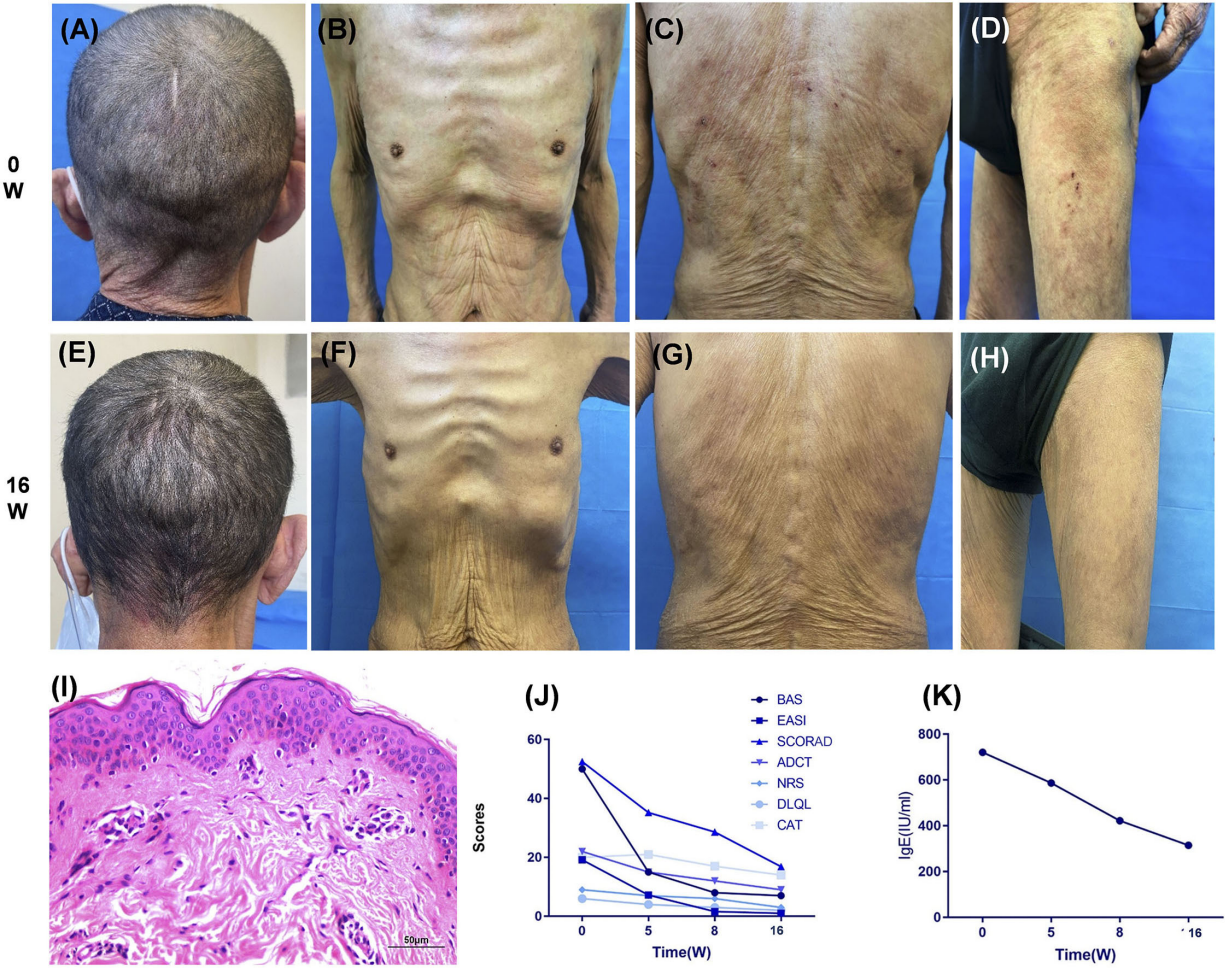
Dupilumab treatment impact on rash, score, and IgE levels over 16 weeks. **(A–D)** Upon initial examination, the patient presented with symmetrical erythema across the head **(A)**, face, trunk **(B, C)**, and proximal extremities **(D)**. Additional symptoms included minor papules, scratch marks on the back and thighs, epidermal defects, and dry skin. **(I)** A skin biopsy from the shoulder revealed mild hyperkeratosis, acanthosis, slight spongiosis, and focal basal cell liquefaction degeneration, indicative of chronic superficial dermal perivasculitis. **(E–H, J, K)** Following a 16-week treatment period with dupilumab, the patient achieved an EASI-90 remission **(J)**, accompanied by significant reductions in multiple atopic dermatitis scores and IgE levels **(K)**. In addition, the CAT score for chronic respiratory diseases showed substantial improvement **(J)**. BAS, body surface area; EASI, Eczema Area and Severity Index; SCORDA, Scoring Atopic Dermatitis Index; ADCT, Atopic Dermatitis Control Tool; NRS, Numerical Rating Scale; DLQL, Dermatology Life Quality Index; CAT, Chronic Obstructive Pulmonary Disease Assessment Test.

A 71-year-old man presented with recurrent erythematous and papular rash associated with pruritus for more than 10 years and generalized aggravation of symptoms for 2 years. The patient had chronic obstructive pulmonary disease (COPD) grade 3 and for more than 10 years, pulmonary heart disease for more than 5 years, and required continuous oxygen inhalation under static state. His brother and grandson suffered from allergic rhinitis. He had been repeatedly treated with topical steroids and oral antihistamines for skin diseases. Physical and auxiliary examinations of the patient are shown in **Figure 1**. The diagnosis according to Hanifin and Rajka criteria was AD (severe). Dupilumab (600 mg) was administered subcutaneously, as well as a topical dexamethasone cream, and a follow-up visit was scheduled for 2 weeks later.

**Figure 2:**
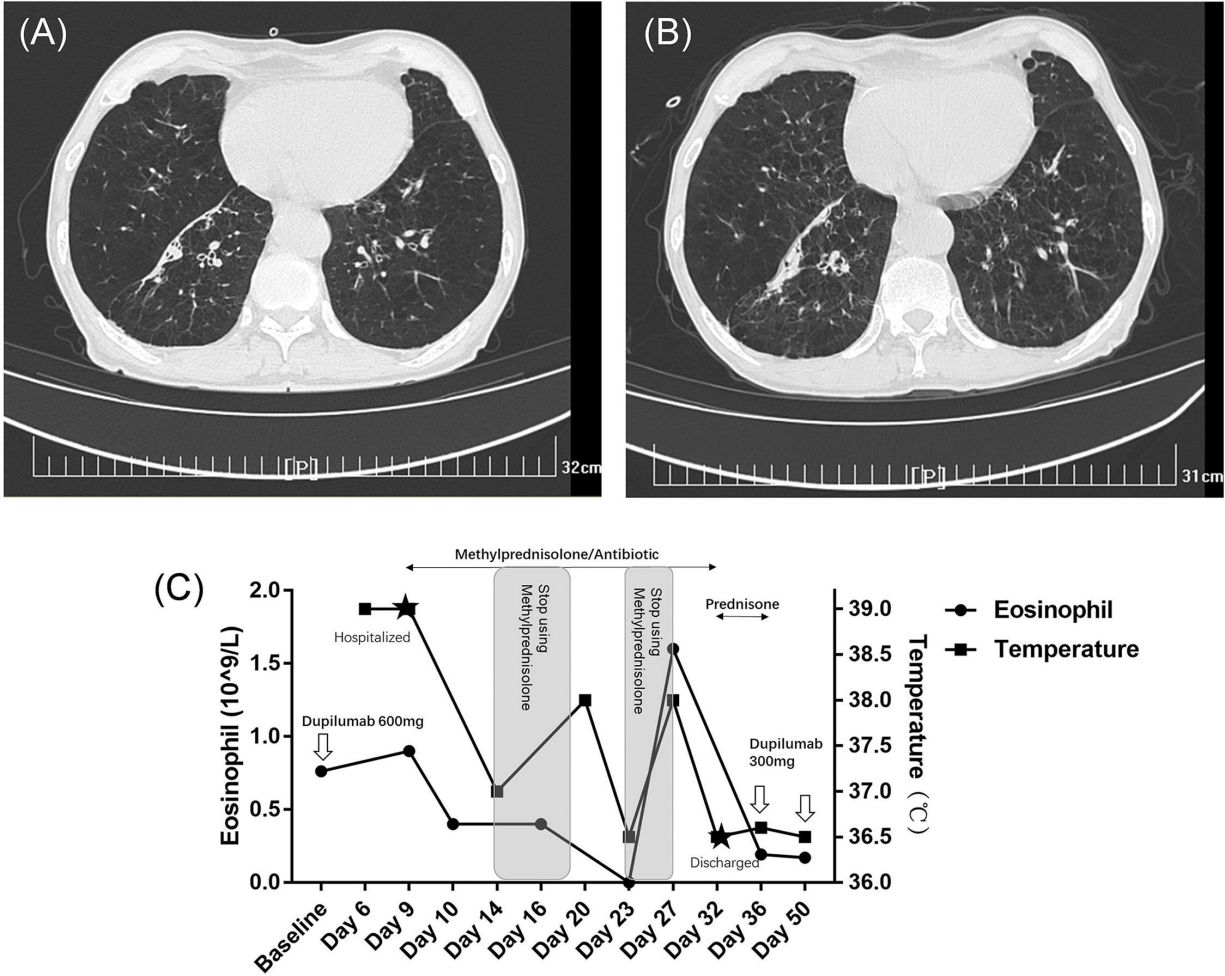
Temperature, EOS, and lung CT changes before, during, and after hospitalization for pulmonary symptoms. **(A)** A lung CT scan conducted 3 months prior to hospitalization revealed a history of bilateral chronic bronchitis and emphysema, along with several old lesions beneath the pleural space of the right lower lobe. **(B)** On the day of hospitalization, the lung CT scan showed scattered ground-glass opacities in both lungs, more pronounced in the lower lobes, and interlobular septal thickening, suggesting potential mild interstitial pneumonia changes. The old subpleural lesions in the lower lobe remained unchanged. **(C)** Absolute EOS and temperature fluctuations were consistent and responsive to glucocorticoid therapy. Methylprednisolone was administered during hospitalization as the patient’s temperature, cough, and shortness of breath had worsened, and blood eosinophil levels had increased on two separate occasions.

Six days later, the patient presented with a high fever (maximum of 39.5 centigrade), cough, a small amount of white sputum, and increasing shortness of breath. Oxygen saturation was decreased to 90%, and his COPD Assessment Test (CAT) score was 25. He was subsequently hospitalized at a community hospital. During hospitalization, blood gas analysis showed a pO_2_ of 75 mmHg, pCO_2_ of 45.7 mmHg, and HCO_3−_ of 29.0 mmol/L, and lung computed tomography (CT) scans revealed interstitial pneumonia (**Figure 2**). Several times during hospitalization, the patient’s eosinophils (EOS) became elevated (up to 1,600 cells/μl) in parallel with increased body temperature and shortness of breath (**Figure 2**). Sputum, fungal, and bacterial examinations; G test; and serum *Aspergillus* antigen, Epstein–Barr virus, Anti-Neutrophil Cytoplasmic Antibodies (ANCA), and antineutrophil cytoplasmic antibodies tests were negative. Bronchoscopy revealed that the airways were normal, and no neoplasms or mucosal erosions were observed. Lavage fluid examination showed an EOS ratio of 30% and no tumor cells. Metagenomic pathogen detection yielded positive results for human cytomegalovirus 5 and human herpes simplex virus 1, but no other pathogens were detected. Expiratory nitric oxide test results in 30 ppb, which suggested mixed airway inflammation. Methylprednisolone was administered during hospitalization as the patient’s temperature, cough, and shortness of breath worsened again on two occasions of intermediate discontinuation. The patient was diagnosed with Acute Eosinophilic Pneumonia (AEP), and symptoms were effectively controlled after 20 days of treatment, with body temperature returning to normal, oxygen saturation stabilizing to above 98%, and CAT score improving to 17. The patient was discharged from the hospital and continued to take oral prednisone (30 mg qd) with a dose reduction of 10 mg every 3 days.

The patient followed up with dermatology 3 days after discharge (5 weeks after dupilumab injection). The rash was significantly relieved, and pruritus scores were improved. After full communication, the patient insisted on continuing dupilumab for his AD. He signed the relevant informed consent form and was willing to provide medical records and pictures. Following continued treatment with dupilumab (300 mg; iH q2w), his rash, pruritus score, and immunoglobulin (Ig) E levels gradually decreased. Furthermore, EOS remained within the normal range, and his CAT score also decreased. By the return visit at 16 weeks, the patient had reached eczema area and severity index (EASI)–90 (**Figure 1**). Until more than 10 months of follow-up, the patient still did not experience any recurrence of EP or rash.

## 3 Literature review of dupilumab-related EP cases

A total of nine articles reporting on 12 cases of dupilumab-related EP were identified and analyzed using the MEDLINE/PubMed and Web of Science Core Collection databases (**Table 1**) (10–18). Specific retrieval terms included: (dupilumab) or (IL-4Rα) or (IL-4/IL-13) AND “Eosinophilic Pneumonia” or “EP” or “Pneumonia.” Including our own case, there were 13 patients in total, consisting of four men and nine women, aged between 37 and 71 years (mean age: 56.46 ± 9.25). There were two patients under the age of 45, seven patients between 45 and 59 years, and four patients aged 60 or above. Of these 13 patients, 11 had pulmonary diseases (either being treated with dupilumab or as comorbidities), including 10 cases of asthma and one case of COPD. Seven patients had rhinitis. Six patients had only pulmonary diseases, five patients had both pulmonary diseases and rhinitis, and two patients had only rhinitis. Among them, six patients were non-smokers, and only one patient had a history of smoking. Past treatment history was available for 10 patients, with only two patients not having received systemic or inhaled corticosteroids. The remaining patients had received inhaled corticosteroids (four cases), oral corticosteroids (two cases), or both (two cases).

**Table 1:** Clinical profiles of eosinophilic pneumonia patients after dupilumab use in the literature.

No.	Sex/Age	Dupilumab use reason	Steroid History	Comorbidity	Smoker	Time to pneumonia after dupilumab	Eosinophils (BAL/Sputum)	IgE changes (IU/mL)	EOS changes (cells/μL)	Treatment and prognosis
1 (Devaraj et al., 2017)	58/M	Asthma	N/A	N/A	N/A	6 weeks	N/A	Normal	3,200 (pneumonia)	Prednisone
2 (Adunse et al., 2021)	55/F	AD BP	N/A	Asthma	N/A	10 weeks	29	2096 (pneumonia)	17,000 (pneumonia)	Methylprednisone of 60 mg and then prednisone of 40 mg daily
3 (Menzella et al., 2019)	56/M	Asthma	Inhaled Steroids	CRNP	No	16 weeks	60	406.9 (baseline)	660 (baseline) | 1,840 (4 weeks) | 2,800 (pneumonia)	Initiated PSL of 50 mg, 6-month course, good prognosis
4 (Sudo et al., 2022)	65/F	ECRS	No steroid use	N/A	N/A	8 weeks	70	–	1,700 (pneumonia)	PSL of 30mg/day, combined 10 mg of steroids and dupilumab for 10 months
5 (Nishiyama et al., 2022)	37/F	Asthma ECRS	Inhaled and Oral steroids	N/A	No	5 months	N/A	674(baseline)| 147 (pneumonia)	276 (naseline) | 9,040 (pneumonia)	PSL of 30 mg/day
6 (Nishiyama et al., 2022)	40/F	Asthma ECRS	Inhaled and Oral steroids	N/A	Ex	3 months	N/A	369(baseline)| 172 (pneumonia)	520 (baseline) | 5,250 (pneumonia)	PSL of 30 mg/day
7 (Kurihara et al., 2022)	55/F	ECRS	Inhaled steroids	Asthma	No	5 weeks	29	N/A	3,852.8 (pneumonia)	PSL of 50 mg/day, tapered
8 (Kurihara et al., 2022)	59/M	ECRS	Inhaled steroids	N/A	No	11 weeks	25.5	N/A	4850 (pneumonia)	PSL of 0.5 mg/kg/day, relapse after tapering, restarted steroids
9 (Gharaibeh et al., 2022)	66/F	Asthma	N/A	N/A	N/A	1 month	31	N/A	1,403 (baseline) | 6,100 (pneumonia) | 11,232 (peak)	Prednisone of 60mg/day
10 (Kanata et al., 2023)	63/F	AD	No systemic steroid use	Asthma	No	4 days	50	2.4 (pneumonia)	610 (baseline) | 1,770 (pneumonia) | 7,360 (peak)	PSL of 30 mg/day, 124 days, no recurrence after stop
11 (Frohlich et al., 2023)	51/F	Asthma	Oral steroids	N/A	N/A	4 months	60 (sputum)	N/A	270 (baseline) | 760 (pneumonia)	50 mg of prednisone
12 (Frohlich et al., 2023)	58/F	Asthma	Oral steroids	CRNP	N/A	12 weeks	N/A	N/A	200 (baseline) | 24,600 (pneumonia)	Prednisone of 60mg/day, symptoms relieved after 4 months

BAL, bronchoalveolar lavage fluid; EOS, eosinophils; IgE, immunoglobulin E; CRNP, chronic rhinosinusitis with nasal polyps; ECRS, eosinophilic chronic rhinosinusitis; PSL, prednisolone; AD, atopic dermatitis; BP, bullous pemphigoid; N/A, not applicable; COPD, chronic obstructive pulmonary disease.

The onset of symptoms after starting dupilumab ranged from 4 days to 5 months, with 11 cases occurring after more than 1 month. The shortest onset times of 4 days and 6 days occurred in patients using dupilumab for AD. Among the 12 literature cases, only Case 3 in **Table 1** provided specific dosage information (300 mg biweekly), whereas Cases 1 and 11 mentioned “biweekly dupilumab” without specifying the dosage. The remaining cases did not specify the exact dosage, frequency, or specific details of use. EOSs in bronchoalveolar lavage fluid ranged from 25.5% to 70%. Among the seven patients with complete Ig E data, four patients showed a decrease or normal levels after the onset of disease. However, all patients showed a significant increase in blood EOSs, regardless of whether their initial EOS count was within the normal range. There was no correlation between the peak EOS count and the severity of fever or respiratory failure.

After systemic corticosteroid treatment, EOS counts decreased, body temperature returned to normal, and respiratory symptoms improved. Prednisone or prednisolone was generally used at a dose of 0.5 mg/kg body weight. The duration of corticosteroid use varied from 3 months to 10 months, whereas our patient only required 1 month of treatment.

Only one patient in the literature (11), along with our patient, resumed dupilumab therapy. The patient in the literature used it in combination with 10 mg of prednisone for 10 months without recurrence of pneumonia. In our case, the patient overlapped corticosteroid use for half a month and then continued dupilumab alone for 9 months without recurrence of pneumonia.

## 4 FAERS database analysis of dupilumab-related EP cases

A total of 110 cases of EP associated with the use of dupilumab were identified from AE reports in the FAERS database, covering the period from 2017 to 2023. After excluding 17 cases with unclear reasons for use, we focused our analysis on the remaining 93 cases, as presented in **Table 2**. Of these 93 cases, age was not reported in 17 cases. Among the remaining 76 cases, the average age was 57.13 ± 15.41 years, ranging from 17 to 82 years. There was one case in the 17-year-old age group, 11 cases in the 18–44 age group, 30 cases in the 45–59 age group, and 34 cases in the 60 and above age group. Regarding gender distribution among the 93 cases, 14 were unspecified, 38 were men, and 41 were women.

**Table 2:** Clinical data of 93 dupilumab-related eosinophilic pneumonia cases in the FAERS database.

Characteristics	Value (n = 93)
Sex, n (%)	
Male	38 (40.8)
Female	41 (44.1)
Missing	14 (15.1)
Age (Years), n (%)	
Age<18	1 (1.1)
18 ≤ Age<45	11 (11.8)
45 ≤ Age<60	30 (32.3)
60 ≤ Age	34 (36.6)
Missing	17 (18.2)
Outcome, n (%)	
Hospitalized	45 (48.4)
Life-threatening	6 (6.5)
Disability	1 (1.1)
Diseases, n (%)	
Lung disorder^*^	37 (39.8)
Rhinitis^**^	32 (34.4)
Lung disorder with rhinitis	19 (20.4)
No lung disease or rhinitis	5 (5.4)

*Lung disorders: asthma, bronchitis, pneumonia, etc.

**Rhinitis: eosinophilic rhinitis, rhinitis with nasal polyps, chronic rhinitis, etc.

Among these 93 cases, 55 had pre-existing lung conditions such as asthma, bronchitis, or pneumonia. In addition, 49 cases had rhinitis. There were 37 cases with only lung disease, 32 cases with only rhinitis, and 19 cases with both conditions. Only five of the 93 cases did not have any lung disease or rhinitis and were diagnosed with AD or eczema.

Of the 93 cases, 92 were classified as severe AEs. Forty-five cases required hospitalization, six of which posed a life-threatening condition, and one case resulted in functional impairment.

## 5 Discussion

Eosinophilic pneumonia (EP) is a disease characterized by eosinophilic infiltration of the lung parenchyma, with categorization varying based on primary or secondary causes (6, 19). The most common drug factors include antibiotics such as daptomycin, minocycline, and nitrofurantoin, which frequently result in chronic EP (6). With the advent of new therapeutic drugs, there has been an increase in reports of EP induced by immunotherapeutic drugs, including immune checkpoint inhibitors, immunomodulators, and biological targeted drugs (20–23). However, overall, EP caused by the dupilumab monoclonal antibody is not common, with isolated reports of dupilumab being used to treat CEP (19, 24).

In our case, the patient was diagnosed with AEP due to acute onset and significant respiratory failure. The patient had no history of smoking and did not use any new drugs except for dupilumab in the past 6 months. Moreover, he had been living at home for a long time due to COPD and pulmonary heart disease, ruling out the common environmental inducements of AEP. Therefore, his AEP was related to the use of dupilumab.

In the literature review, we explored various indications for dupilumab. Notably, two cases involving patients with AD exhibited EP shortly after the first dose, whereas the remaining cases developed symptoms after at least two administrations of dupilumab. With limited detailed case descriptions available in the current literature, the precise association between the occurrence of EP and the loading dose or cumulative dosage remains elusive. Specifically, in the context of adult AD, the standard dosage is 300 mg every 2 weeks, with an initial loading dose of 600 mg. However, the necessity of the loading dose for individuals at high risk of EP AEs is currently inadequately addressed in the literature, with only two reported cases. This highlights the need for future investigations to provide more comprehensive insights into the relationship between EP occurrence, dosage patterns, and potential considerations for high-risk populations.

Through a literature review and analysis of the FAERS database, we found that the incidence rate is highest among individuals aged 45–60 and over 60. Most patients have a history of respiratory diseases, primarily lung diseases. Most present changes of CEP, and all patients show elevated EOS, peaking during pneumonia episodes. Despite the severe presentation of EP, moderate to high doses of steroid treatment can achieve excellent results. Currently, there are few reports on whether to restart dupilumab treatment. Only our patient and another case restarted dupilumab treatment after controlling pneumonia. Regardless of whether steroids were used concurrently, no recurrence or worsening of pneumonia was observed. However, this still requires more data support.

Dupilumab, an inhibitor of T2-type inflammatory cytokines IL-4 and IL-13, indirectly impacts IL-5 production. Mechanistically, it inhibits the differentiation and growth of EOS (25, 26). However, 1% to 9% of patients with AD experience transient elevations of EOS after treatment with dupilumab (26, 27). Several factors may contribute to this phenomenon. Dupilumab can suppress the expression of vascular cell adhesion protein 1 (VCAM-1), chemokines, and periostin, as well as the secretion of chemotactic factors through IL4 and IL-13 (12 ,16, 28, 29). Consequently, EOSs that fail to infiltrate tissues might temporarily surge in the bloodstream. This peripheral blood eosinophilia may be associated with organ damage, including conjunctiva and lungs (14). Studies have shown that patients with dupilumab-related conjunctivitis and AD showed a significant increase in peripheral blood EOS count (30). Furthermore, all instances of dupilumab-associated EP with comprehensive lung biopsy revealed eosinophilic infiltration in the lungs, suggesting mechanisms facilitating EOS migration to the lungs. These variations might be attributed to diverse eosinophilic infiltration mechanisms, such as VCAM-1 expression induced by non–IL-4/13 pathways, and translocation through non-VCAM-1 EOS adhesion mechanisms (12). This allows EOS extracellular trap death to be seen in nasal polyp tissues but not in lung tissues (12). Our literature review and database analysis indicate that most patients have a history of lung disease. This raises the question: Could underlying lung disease, in the context of lung injury, trigger EOS migration to the lungs via these pathways?

The potential involvement of IL-5 in the mechanism of dupilumab-related EP adds another layer of complexity to our understanding. The generation process of airway EOSs not only is regulated by type 2 cytokines, IL-4 and IL-13, but also involves other cytokines such as IL-33, Thymic Stromal Lymphopoietin (TSLP), and IL-25 due to the engagement of hematopoietic stem cells and progenitor cells (31, 32). This multifaceted regulation makes the EOS dynamics exceedingly intricate. Nishiyama et al. observed elevated IL-5 levels in the blood of pneumonia patients, whereas it remained normal in non-pneumonia patients (12). Despite IL-5, IL-4, and IL-13 all being type 2 inflammatory factors, their interplay has been found to be complex in animal studies. Interestingly, low levels of IL-4 or IL-13 may paradoxically stimulate the production and mobilization of EOSs via IL-5 (33). Although dupilumab does not directly affect IL-5, by blocking IL-4 and IL-13, it may alter the regulation of EOSs, making the effect of IL-5 more prominent and further promoting the accumulation of EOSs in the lungs (10). However, the source of IL-5 or the correlation between blood eosinophilia and lung eosinophilic infiltration remains elusive.

Another possible reason is the steroid-sparing effect of dupilumab, which may reveal EP after discontinuation of oral corticosteroids, rather than being a direct consequence of dupilumab itself (10, 16). Our literature review reveals that 80% of patients had used steroids either inhaled or systemically. Some studies suggest that dupilumab facilitates the withdrawal of oral glucocorticoids, possibly unmasking previously suppressed eosinophilic diseases, such as eosinophilic granulomatosis with polyangiitis. However, in these cases, such scenarios were eventually dismissed.

Based on our case and database analysis, we advocate for vigilant monitoring of EP during dupilumab treatment, particularly in patients over 45 years old, with pre-existing respiratory diseases, especially lung diseases, and those who have undergone orally/inhaled steroid therapy. It is crucial to closely observe changes in peripheral blood EOSs during treatment follow-up. If there is a persistent increase accompanied by symptoms like fever, cough, and difficulty breathing, then EP should be suspected. Early diagnosis and proactive treatment yield favorable outcomes. However, given the current data quality, additional observations are required to ascertain the safety of restarting dupilumab treatment, and more fundamental/clinical research studies are needed to elucidate the relationship between the IL-4/IL-13 pathway and the onset of EP.

## Data availability statement

The original contributions presented in the study are included in the article/supplementary material. Further inquiries can be directed to the corresponding author.

## Ethics statement

The studies involving humans were approved by the Sichuan Academy of Medical Science and Sichuan Provincial People’s Hospital in Chengdu, China (Protocol 2022-327). The studies were conducted in accordance with the local legislation and institutional requirements. Written informed consent for participation in this study was provided by the participants’ legal guardians/next of kin. Written informed consent was obtained from the individual(s), and minor(s)’ legal guardian/next of kin, for the publication of any potentially identifiable images or data included in this article.

## Author contributions

XYZ: Conceptualization, Writing – original draft. GY: Investigation, Software, Writing – original draft. XMZ: Investigation, Formal Analysis, Writing – original draft. LW: Data curation, Validation, Writing – original draft. JX: Visualization, Writing – original draft. JZ: Methodology, Supervision, Writing – original draft. XC: Formal Analysis, Writing – review & editing. LZ: Project administration, Writing – review & editing.
